# Chronic infection with *Mycobacterium lepraemurium* induces alterations in the hippocampus associated with memory loss

**DOI:** 10.1038/s41598-018-27352-x

**Published:** 2018-06-13

**Authors:** Enrique Becerril-Villanueva, María Dolores Ponce-Regalado, Gilberto Pérez-Sánchez, Alberto Salazar-Juárez, Rodrigo Arreola, María Elizbeth Álvarez-Sánchez, Mario Juárez-Ortega, Ramcés Falfán-Valencia, Rogelio Hernández-Pando, Jorge Morales-Montor, Lenin Pavón, Oscar Rojas-Espinosa

**Affiliations:** 1 Department of Psychoimmunology, National Institute of Psychiatry “Ramón de la Fuente”, Calzada México-Xochimilco 101, Colonia San Lorenzo Huipulco, Tlalpan, 14370 Mexico City Mexico; 2 Departamento de Clínicas, Centro Universitario de los Altos, Universidad de Guadalajara, Tepatitlán de Morelos, Jalisco, Mexico; 3 Branch Clinical Research. Laboratory of Molecular Neurobiology and Neurochemistry of Addiction, National Institute of Psychiatry “Ramón de la Fuente”, Calzada México-Xochimilco 101, Colonia San Lorenzo Huipulco, Tlalpan, 14370 Mexico City, Mexico; 4 Psychiatric Genetics Department, National Institute of Psychiatry “Ramón de la Fuente”, Clinical Research Branch, Calzada México-Xochimilco 101, Colonia San Lorenzo Huipulco, Tlalpan, 14370 Mexico City, Mexico; 5 Posgrado en Ciencias Genómicas, Universidad Autónoma de la Ciudad de México (UACM), San Lorenzo # 290, Col. Del Valle, CP 03100 México City, Mexico; 6 Departamento de Inmunología, Escuela Nacional de Ciencias Biológicas, Instituto Politécnico Nacional, Carpio y Plan de Ayala, Colonia Santo Tomás, 11340 Ciudad de México, Mexico; 7 HLA Laboratory, Instituto Nacional de Enfermedades Respiratorias Ismael Cosío Villegas, Tlalpan 4502, Sección XVI, Tlalpan, 14080 Mexico City, Mexico; 8 Experimental Pathology Section, Pathology Department, National Institute of Medical Sciences and Nutrition Salvador Zubiran, Vasco de Quiroga 15, Colonia Belisario Dominguez Seccion XVI, 14080 Tlalpan, México City Mexico; 9 Departamento de Inmunología, Instituto de Investigaciones Biomédicas AP 70228, México, DF 04510 Mexico

## Abstract

Murine leprosy, caused by *Mycobacterium lepraemurium* (*MLM*), is a chronic disease that closely resembles human leprosy. Even though this disease does not directly involve the nervous system, we investigated a possible effect on working memory during this chronic infection in Balb/c mice. We evaluated alterations in the dorsal region of the hippocampus and measured peripheral levels of cytokines at 40, 80, and 120 days post-infection. To evaluate working memory, we used the T-maze while a morphometric analysis was conducted in the hippocampus regions CA1, CA2, CA3, and dentate gyrus (DG) to measure morphological changes. In addition, a neurochemical analysis was performed by HPLC. Our results show that, at 40 days post-infection, there was an increase in the bacillary load in the liver and spleen associated to increased levels of IL-4, working memory deterioration, and changes in hippocampal morphology, including degeneration in the four subregions analyzed. Also, we found a decrease in neurotransmitter levels at the same time of infection. Although *MLM* does not directly infect the nervous system, these findings suggest a possible functional link between the immune system and the central nervous system.

## Introduction

Neuroimmunoendocrine communication involves an intricate network of interactions between the three major homeostasis systems: neural, immune, and endocrine. Cytokines, hormones, neuropeptides, and their receptors participate in the cross-talk between these systems and their imbalance leads to a wide repertoire of diseases^1–3^. Although the participation of these mediators in the function of these systems is well known, the exact interactive mechanisms and their direct or indirect consequences are not fully understood. For instance, it is not clear how peripheral chronic infection and inflammation lead to injury in the central nervous system (CNS); similarly, the behavioral consequences of these two actions are yet to be elucidated^4–6^. Chronic inflammation is produced by a variety of intracellular microorganisms, including pathogenic mycobacteria, for which different tissues are target organs, resulting in a variety of diseases depending on the mycobacteria; e.g., lung in tuberculosis (*Mycobacterium tuberculosis*) and skin in leprosy (*M*. *leprae*) and Buruli ulcera (*M*. *ulcerans*)^7,8^. In addition to the local tissue damage produced by a wide variety of soluble mediators, the chronicity of the diseases leads to several systemic alterations that may affect the immune, endocrine, and nervous systems (*e*.*g*. anergy, hypercholesterolemia, and neurodegeneration, respectively)^9–11^. These long-term systemic alterations have been described in natural and experimental models of infection^12,13^.

Murine leprosy is an interesting model of a chronic infectious disease that keeps some resemblance with human leprosy^14,15^, whose depressive or somatoform disorders have been documented since ancient times. Lepromatous leprosy patients suffer psychological and psychiatric disorders that have been considered the result of social and auto stigma factors^16,17^, yet might be a probable and direct effect of the infection on mental health. Leprosy itself is the probable cause of mental distress, without the cognitive involvement of the patient; indeed, this might be difficult to prove in the human being, whose conscience will always operate. However, some reports indicate that mice infected with *M*. *bovis* (BCG) exhibit early sickness behavior symptoms^18–24^, including depression-like anomalies that resulted in the lowering of the sucrose ingestion preference test, locomotor activity, and lessened mobility in the forced swim and tail suspension tests.

The study on the balance in pro- and anti-inflammatory cytokines (Th1/Th2) helps to understand the control of infections and the injury mechanisms of infected tissues^7^ and can also provide information on behavioral changes and cognitive impairment associated to infections. Indeed, the loss of cellular immune competence facilitates the disease progression that might reach the CNS, promoting exacerbated neuroinflammation and cognitive impairment, including memory and anxiety. These alterations are strongly associated to the production of neurotoxic components involved in progressive neuronal death in different brain regions, the hippocampus being one of the most affected^4,25^.

Even though it has already been shown that *MLM* does not directly infect the CNS, its chronic systemic distribution might indirectly affect the brain through soluble mediators and subsequently produce behavioral disorders and learning alterations.

Sickness behavior refers to behavioral changes developed in sick individuals during the early phases of a short-term infection and it lasts until the conclusion of the disease. In chronic infections, however, sickness behavior continues until the final stage of the disease. Because of this, chronic infection with *MLM* seems to be an appropriate model for the study of long-term sickness behavior in the mouse.

## Results

### Histopathological changes in liver and spleen of *MLM*-infected mice

**Figure 1:**
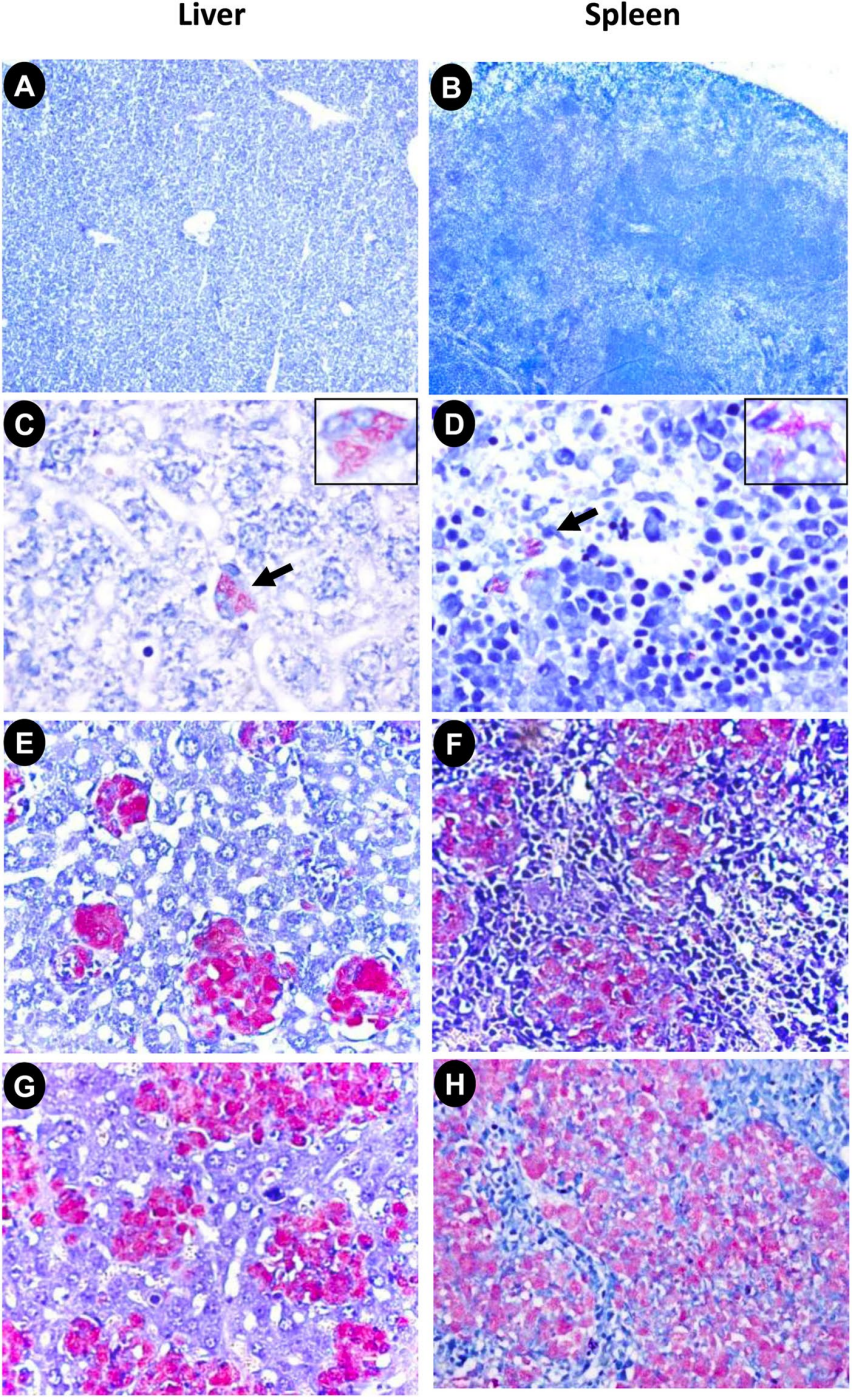
Representative micrographs of liver and spleen of control mice and animals infected with *Mycobacterium lepraemurium* (*MLM*). Liver (**A**) and spleen (**B**) sections from control animals show normal histology. Micrograph correspond to sections of liver and spleen from a mouse at 40 days PI (**D**). Scarce *MLM* in the cytoplasm of macrophages (arrows); some macrophages appear vacuolated and contain bacilli (inset). Liver (**E**) and spleen (**F**) sections from a mouse at 80 days PI show numerous macrophages (globi) with abundant *MLM*. Liver (**G**) and spleen (**H**) sections from a mouse at 120 days PI, large and coalescent granulomas substitute extensive areas of the liver and spleen (H/E, Ziehl-Neelsen stains, all micrographs X40, inset X400 magnification).

Infection with *MLM* produces a chronic granulomatous disease in susceptible mouse strains such as BALB/c. In the present study, inoculation of 20 × 10^6^ bacilli by intravenous route gave rise to progressive granulomatous lesions in liver and spleen parenchyma, with a concomitant steady increase in the number of bacilli (Fig. 1). Mice in the control group did not present morphological alterations in these organs (Fig. 1A,B). By day 40 post-inoculation, early granulomas formed by mononuclear cells (macrophages and lymphocytes) were clearly evident, although they showed scanty acid-fast bacilli by Ziehl-Neelsen staining (Fig. 1C,D). By day 80 post-infection (PI), granulomas expanded and tended to confluence with progressive increase of bacillary loads. At this time, granulomas made-up of bacilli-loaded macrophages were large lesions that replaced most of the liver and spleen parenchyma (Fig. 1E,F). By day 120, the granuloma fraction completely substituted the liver and spleen parenchyma and bacilli became uncountable (Fig. 1G,H).

### Bacillary load in liver and spleen

**Figure 2:**
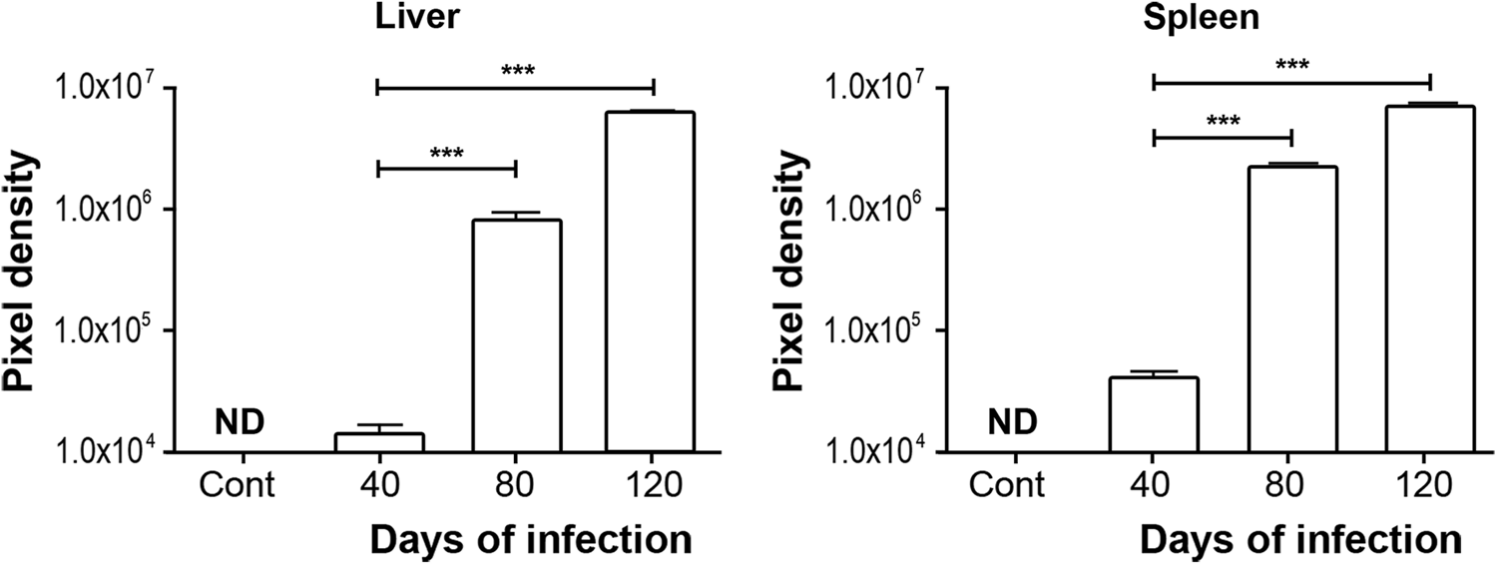
Semi-quantitative assessment of mycobacterial load in liver and spleen of mice infected with *MLM*. Bacillary load (in pixels) in liver (upper panel) and spleen (lower panel) from mice, at 0, 40, 80, or 120 days infected by *MLM*. Values are presented as mean ± SEM. Two-way ANOVA test and *post hoc* Tukey multiple comparisons test. ^***^p < 0.001 as compared to control group.

The semi-quantitative assessment of the bacillary load in each group of mice is illustrated in Fig. 2. Quantitation of bacilli was achieved using IMAGE J software and the units were given in pixels^12^. Measurements were performed on 4-μm paraffin sections stained for acid-fast bacilli (Ziehl-Neelsen). Results correspond to the analysis of 5 independent fields per tissue section. Figure 2A shows the bacillary load in liver (*F*_(492.4) = 32,2_ p < 0.0001). In the *MLM*-infected group, the bacillary load was 1.4 × 10^04^ ± 2.3 × 10^03^ pixels at day 40; 8.2 × 10^05^ ± 1.1 × 10^05^ at day 80, and 5.9 × 10^06^ ± 3.8 × 10^05^ at day 120 post-inoculation (PI). Figure 2B shows the spleen (F_(250.6) = 38,2_ p < 0.0001) with similar trends of bacillary load: 4 × 10^04^ ± 4.5 × 10^03^ pixels at day 40 of infection; 2 × 10^06^ ± 2.1 × 10^05^ at day 80, and 6.7 × 10^06^ ± 5.6^05^ at day 120 PI. Statistical comparison between days 40 and 120 was highly significant (p < 0.0001).

### Cytokine measurement in serum

Serum cytokines were measured using the Luminex® LABScan 100 system (Bead mouse Th1/Th2 6-Plex Panel) (Table 1). IL-2 was not detected in the control group nor in the *MLM-infected* group throughout the entire period of infection (40 to 120 days). In contrast, IL-12 was detected at all infection times, yet, a significant statistical difference compared to the control group was only observed at 120 days of infection (F_(10.51) = 22,3_ p < 0.01). IFN-γ was not detected in the control group nor in the *MLM*-infected groups (40, 80, and 120 days). Type-2 cytokines were not detected in the control group yet the infected animals showed increased levels of IL-4 at post-infection days 80 (49.05 ± 11.17 pg/mL) and 120 (73.99 ± 3.75 pg/mL) (F_(138.8) = 36,3_ p < 0.0001). Interleukin 5 (IL-5) and IL-10 were not detected in any group at any PI time.

**Table 1:** Time-course of serum cytokine levels in mice chronically infected with *Mycobacterium lepraemurium*.

Cytokine	Lower limit of detection(pg/mL)		Days infections *MLM*			Statistical *post hoc* analysis		
		Controls	40	80	120	Cont vs 40	Cont vs 80	80 vs 120
IL-2	5	ND	ND	ND	ND	—	—	—
IL-12	1	7.71 ± 0.78	5.05 ± 0.65	8.44 ± 2.00	14.47 ± 0.20	—	—	*
INF-γ	20	ND	ND	ND	ND	—	—	—
IL-4	10	ND	ND	49.05 ± 11.17	73.99 ± 3.75	—	—	***
IL-5	15	ND	ND	ND	ND	—	—	—
IL-10	15	ND	ND	ND	ND	—	—	—

Quantification of cytokines in sera of control and *MLM*-infected mice. Values are presented as mean ± SEM. Two-way ANOVA test, *post hoc* Tukey multiple comparisons test. *p < 0.05; **p < 0.01 and ***p < 0.001 as compared to control group. IL- = interleukin; ND = no-detectable; IFN = interferon; *MLM* = *Mycobacterium lepraemurium*.

### Immunohistochemical cytokine detection

**Figure 3:**
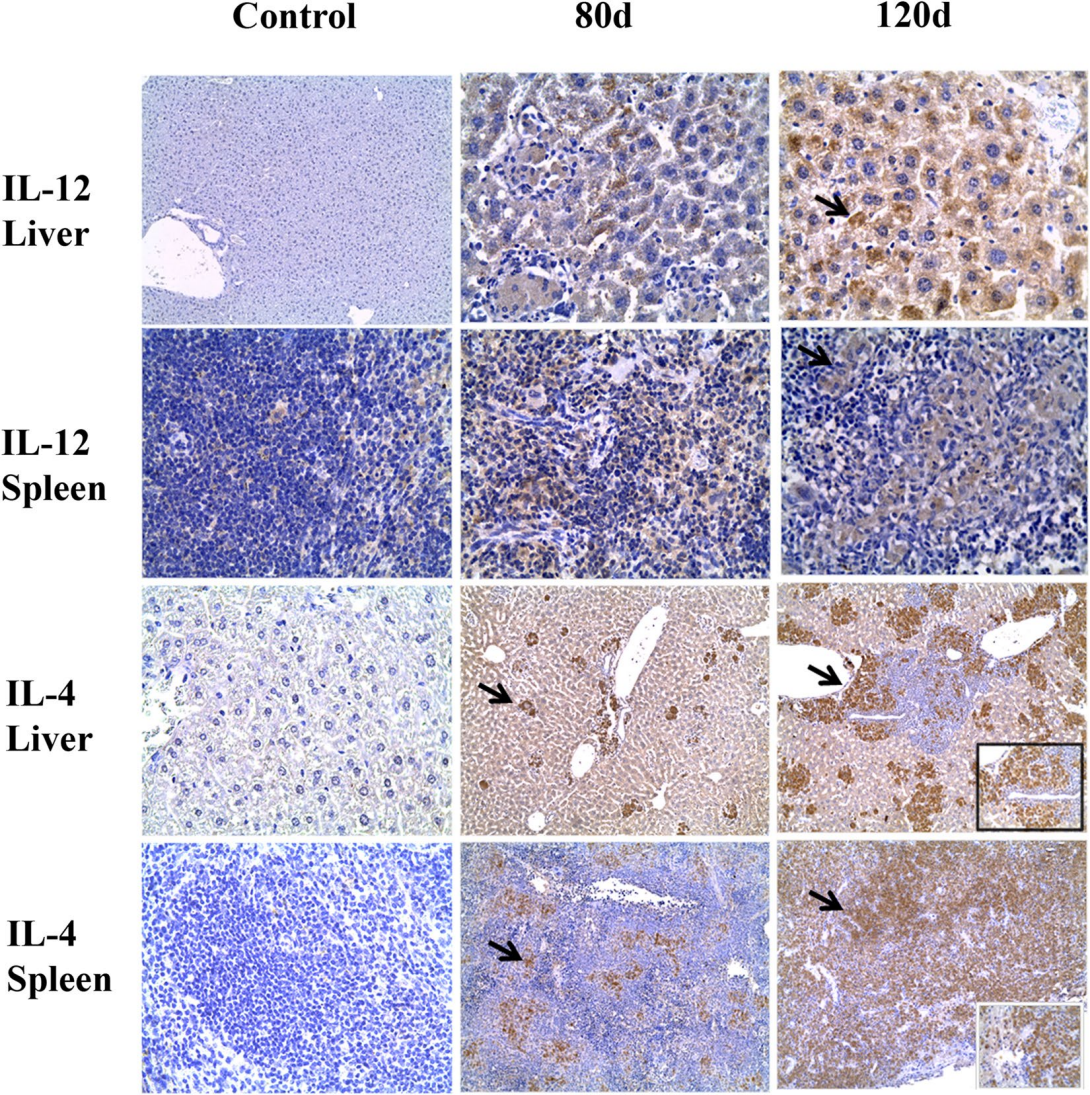
Photomicrographs show immunostaining for IL-12 and IL-4 in liver and spleen. Staining for IL-12 shows focal positivity in splenic macrophages in the control group. At 80 and 120 days PI hepatic parenchyma exhibits focal positivity in Kupffer and multifocal macrophages. In spleen, multifocal staining is observed in macrophages in the splenic white pulp (arrows) X400. IL-4 immunolabeling is negative in the control group but at day 80 PI IL-4 staining is present on incipient granulomas in liver and it appears multifocally distributed in lymphocytes in the splenic red pulp (arrows). Diffuse IL-4 positivity is observed in extensive inflammatory hepatic infiltrates at day 120 PI. Diffuse IL-4 staining is also observed on spleen lymphocytes (box). X200 and X400 micrographs.

In the control group, IL-12 was only detected in macrophages of the splenic white pulp. By day 80 PI, IL-12 was observed in Kupffer cells and clusters of macrophages in the spleen white pulp. By days 80 and 120 PI, diffuse staining was observed in hepatic parenchymal macrophages and the red pulp of the spleen (Fig. 3). By day 80 PI, incipient granulomas were detected in the hepatic parenchyma and staining for IL-4 was observed in isolated and small clusters of lymphocytes. Multifocal IL-4 staining was also observed in lymphocytes of the spleen parenchyma. By day 120 PI, staining for IL-4 was intense mainly in the granuloma lymphoid infiltrate but also in the splenic parenchyma.

### Working memory (T-maze test)

**Figure 4:**
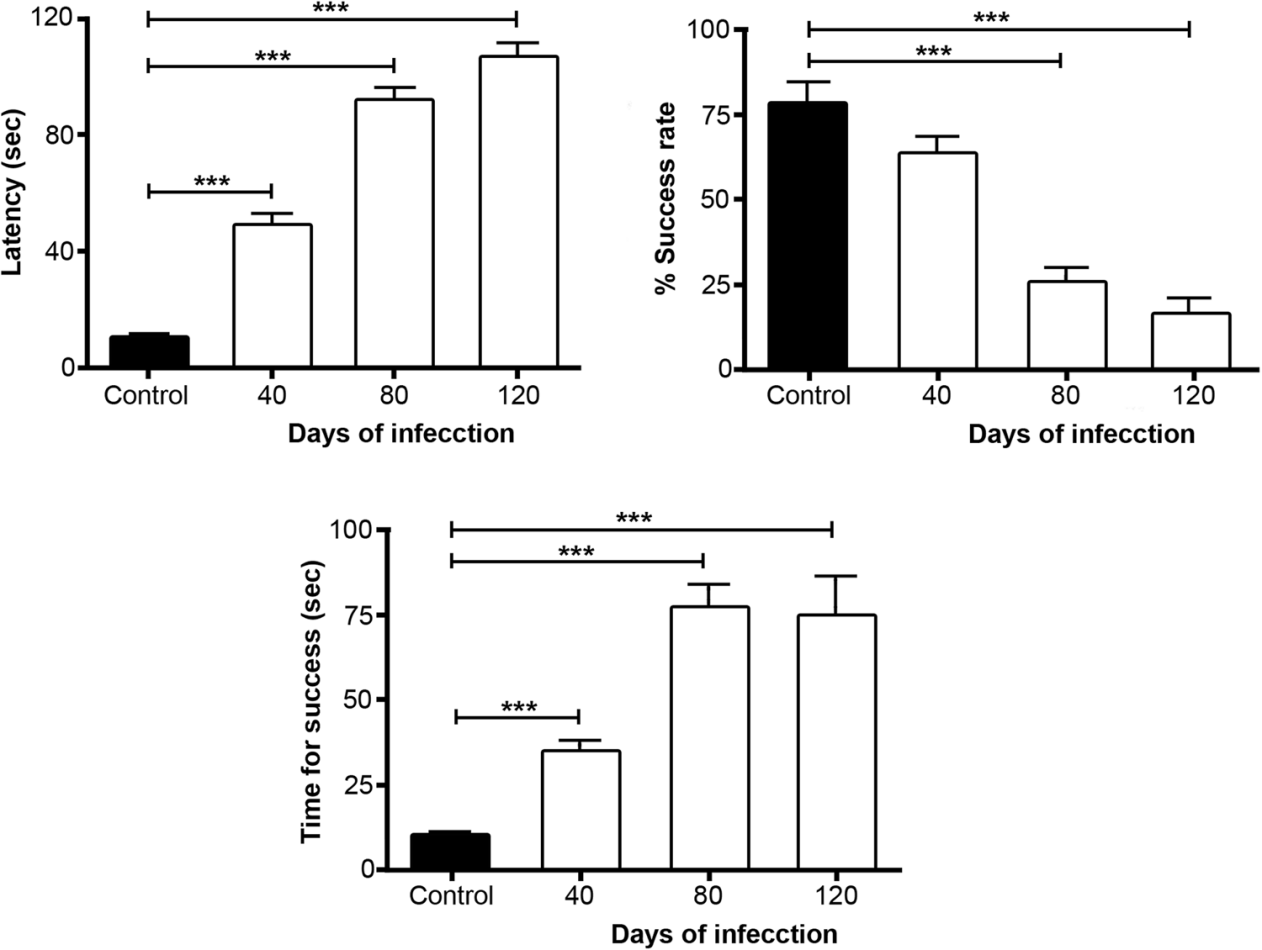
Effect of chronic infection induced by *MLM* on working memory. Time required to fulfill the test (**A**); increase in execution time reflects cognitive deterioration. Percentage of success during 12 tests performed on each mouse (**B**). Time required to successfully execute the test (**C**). Values are presented as mean ± SEM. Two-way ANOVA test, *post hoc* Tukey multiple comparisons test. ^***^p < 0.001 as compared to control group.

For the assessment of working memory, five mice of each group were tested. Each test consisted of 12 sessions with an inter-test period not longer than 2 min; the maximal time-lapse for each test was 120 seconds (sec). Figure 4A illustrates the latency time (sec), *i*.*e*., the time taken by each mouse of each group to complete the test. Latency time was 10.26 ± 1.12 sec in the control group, 48.8 ± 1.12 sec in the 40-day PI group, 92.18 ± 3.63 sec in the 80-day PI group, and 106 ± 4.62 sec in the 120-day PI group. Values at 40-, 80- and 120-PI days greatly differed from the value in the control group (F_(86.59) = 298,3_ p < 0.0001).

To evaluate short-term memory associated with reward, the percentage of attempts leading to a successful hit during the 12 sessions was calculated. The efficiency in reaching the arm with the reward was 78% in the control group, 64.8% in the 40-day PI group, 26.8% in the 80-day PI group, and 16.6% in the 120-day PI group, the last two values being statistically different from the one in the control group (F_(27.81) = 42,3_ p < 0.001) (Fig. 4B).

Time taken to succeed in reaching the award was also calculated (Fig. 4C). Animals in the control group took a shorter time to complete the test compared to the 40-day PI group (9.72 ± 1.18 *vs* 34.19 ± 3.32 sec), the 80-day PI group (76.37 ± 6.73 sec), and the 120-day PI group (73.66 ± 11.56 sec), with differences highly significant (F_(39.95) = 140,3_ p < 0.0001).

### Spontaneous locomotor activity

**Figure 5:**
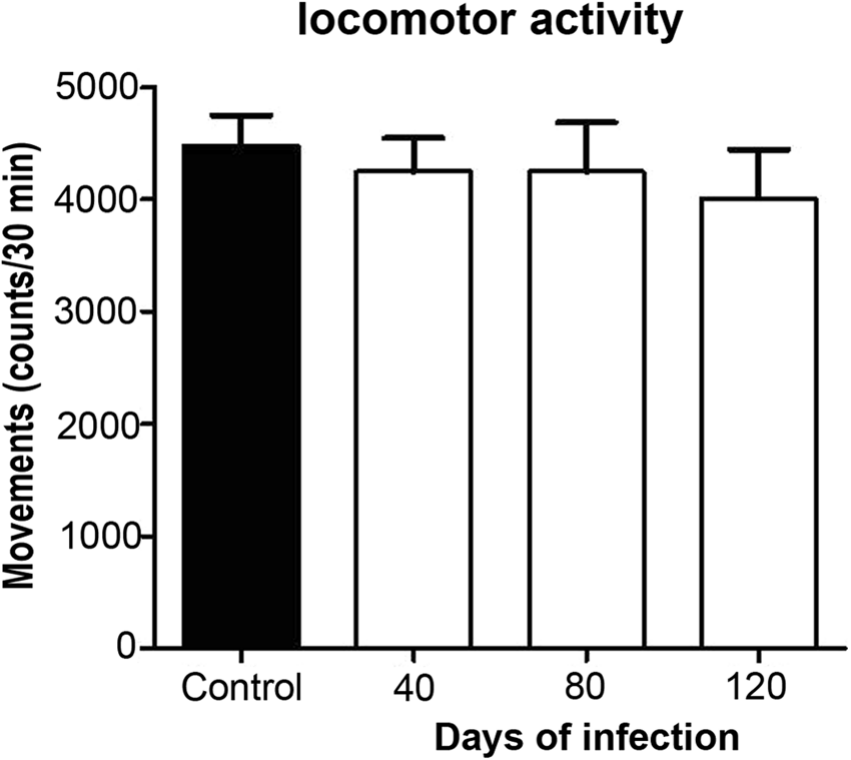
Spontaneous locomotor activity in mice chronically infected with *MLM*. There were no differences in the locomotor activity of mice of any group, regardless the time of infection.

The spontaneous locomotor activity test was applied to the animals of the four groups to detect possible damage in regions other than the hippocampus, *e*.*g*., corpus striatum and basal ganglia^26,27^. No altered locomotor activity was observed in any of the four groups during the 30-min test (Fig. 5).

### Histological study in the hippocampus

**Figure 6:**
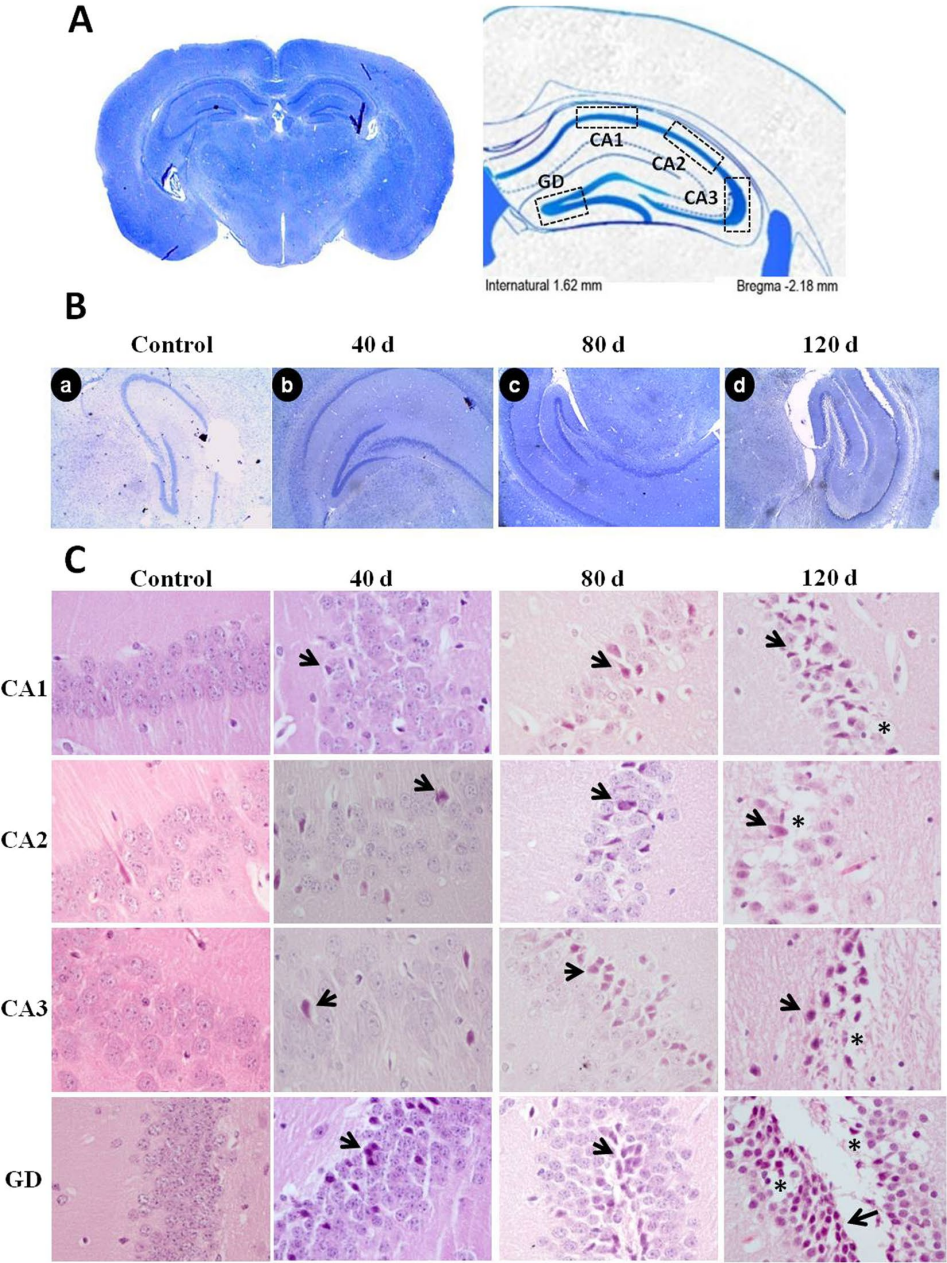
Microphotographs correspond to the hippocampus sections evaluated (**A**). Ziehl-Neelsen staining at 0, 40, 80, and 120 days PI (**B**). No bacillary infiltrate or lesions that indicate gliosis or ischemic processes in brain parenchyma were observed (a,b,c,d) (**C**). Representative micrographs from CA1, CA2, CA3, and GD at 0, 40, 80, and 120 days PI. Cytological changes include neurons with acidophilic cytoplasm and fragmented (arrows) or pyknotic nuclei. These changes were progressive and suggested neurodegenerative damage associated to chronic infection by *MLM*. (H-E, X400).

**Figure 7:**
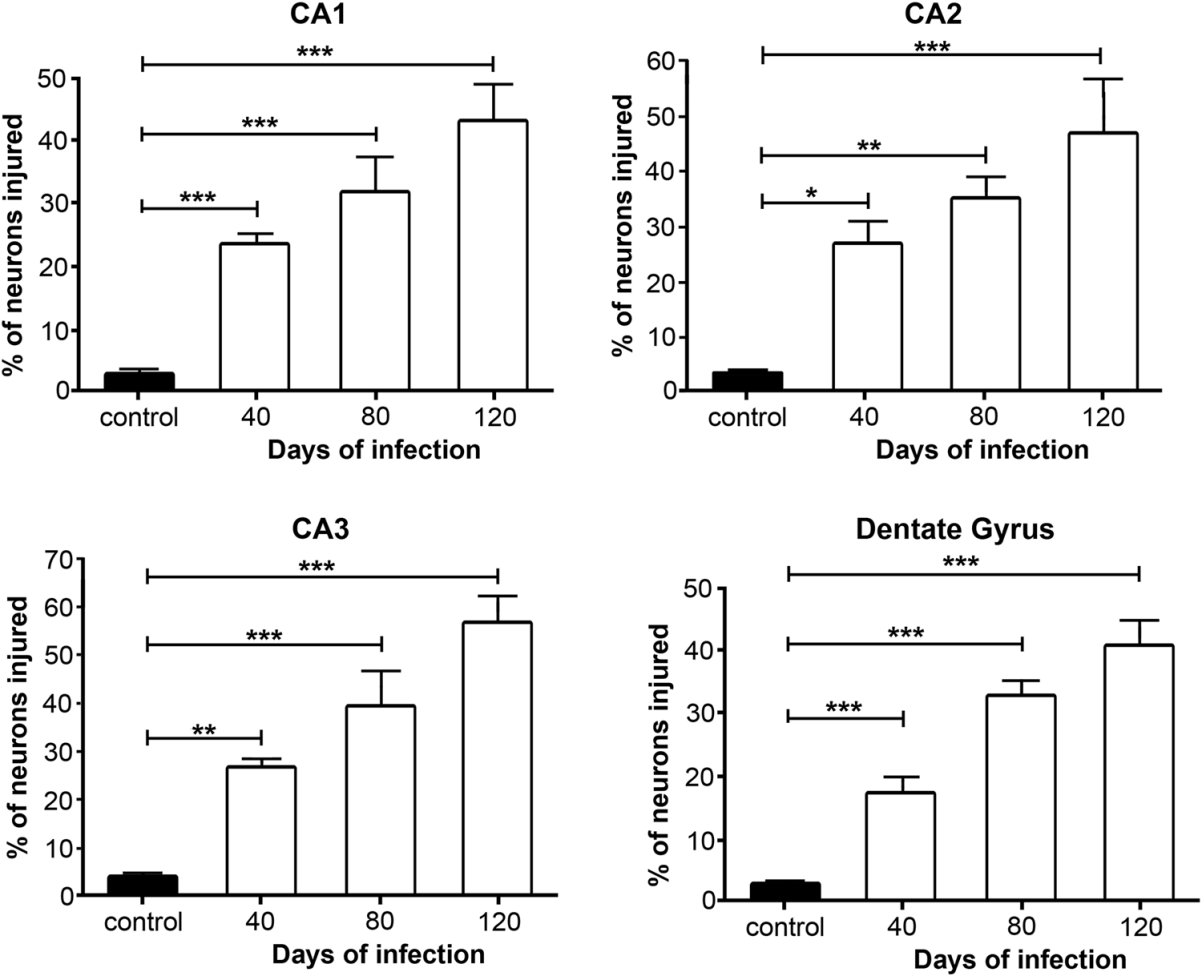
Percentage of injured neurons in hippocampal regions. Five independent fields were analyzed per region and the number of neurons with pyknotic, fragmented, and acidophilic nuclei, and acidophilic cytoplasm was calculated. Data are presented as percent values (Mean ± SEM). Two-way ANOVA test, *post hoc* Tukey multiple comparisons test. ^*^p < 0.05; ^**^p < 0.01 and ^***^p < 0.001 as compared to control group.

To evaluate histological damage in the hippocampus, several parameters were taken into account, particularly those indicative of cell death such as basophilic pyknotic nuclei, acidophilic cytoplasm, and fragmented nuclei in regions CA1, CA2, CA3, and DG (bregma regions −1.82 and −2.46 mm)^28^. The general morphology of hippocampus in the control group was well preserved, with neurons of normal characteristics. By day 40 PI, neurons with pyknotic nuclei and acidophilic cytoplasm were observed in the four regions analyzed. By day 80, the number of pyknotic-acidophilic neurons increased and the thickness of the layer regions decreased. At 120 days PI, these alterations had further progressed. The histologic changes observed in the hippocampus of the *MLM*-infected animals are characteristic of non-specific degenerative processes (Fig. 6B). The most remarkable result that emerged from the analysis was the lack of cell infiltrates and bacilli in the brain parenchyma that might suggest direct microbial infection in the hippocampus (Fig. 6B). The percentage of neurons with morphological alterations indicative of cell damage in CA1, CA2, CA3, and DG, increased progressively and changes were highly significant when compared to the histology in the control group (p < 0.001) (Fig. 7).

### Neurotransmitters quantification by HPLC

**Figure 8:**
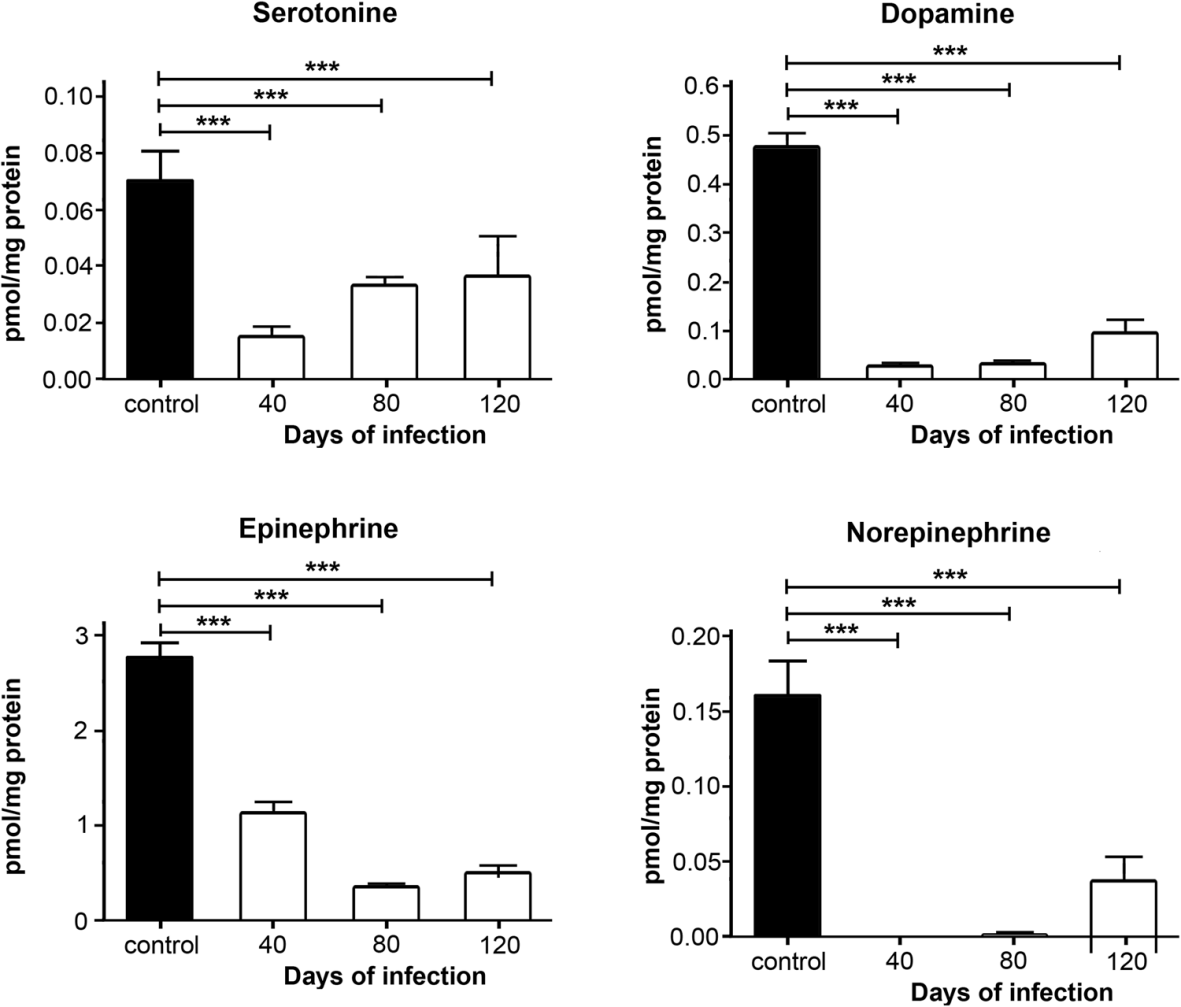
Concentration of diverse neurotransmitters (serotonin, dopamine, epinephrine and norepinephrine) determined by HPLC in hippocampus of animals infected with *MLM*. Values correspond to pmol/mg protein and are presented as mean ± SEM. Two-way ANOVA test, *post hoc* Tukey multiple comparisons test. ^***^p <0 001 as compared to control group.

Figure 8A shows serotonin concentration (5-HT) in the different study-groups. The level of 5-HT was 0.069 ± 0.003 pmol/mg protein in the control group, 0.014 ± 0.001 pmol/mg in the 40-day PI group, 0.033 ± 0.001 pmol/mg in the 80-day PI group, and 0.036 ± 0.005 pmol/mg in the 120-day PI group. Values in the infected groups were significant lower than the value in the control group (F_(39.86) = 23,3_ p < 0.001).

Figure 8B shows the results on the levels of dopamine (DA) in the four study-groups. These were 0.476 ± 0.02 pmol/mg in the control group, 0.026 ± 0.001 pmol/mg in the 40-day PI group, 0.031 ± 0.004 pmol/mg in the 80-day PI group, and 0.094 ± 0.02 pmol/mg in the 120-day PI group. Values in the infected groups were significantly lower than the value in the control group (F_(145.7) = 29,3_ p < 0.001).

Figure 8C depicts the levels of epinephrine (EP) in the four study-groups. These levels were 2.75 ± 0.15 pmol/mg in the control group, 1.12 ± 0.11 pmol/mg in the 40-day PI group, 0.35 ± 0.01 pmol/mg in the 80-day PI group, and 0.5 ± 0.07 pmol/mg in the 120-day group. Values in the infected groups were statistically different from the value in the control group (F_(116.9) = 29,3_ p < 0.001).

Figure 8D shows the levels of norepinephrine (NE) in the four study-groups. These values were 0.16043 ± 0.024 pmol/mg in the control group, 0.0016 ± 0.0009 pmol/mg in the 80-day PI group, and 0.03762 ± 0.015 pmol/mg in the 120-day PI group. NE was not detected in the 40-day PI group. Statistically significant differences were observed between control and *MLM*-infected groups (F_(26.43) = 31,3_ p < 0.001).

## Discussion

The progressive decrease in cell-mediated immunity, CMI (anergy), is characteristic of chronic infectious diseases produced by pathogenic mycobacteria^12,29,30^. Here, we showed that infection of mice with *Mycobacterium lepraemurium* (*MLM*) led to a disseminated, multiorgan, infection that did not directly affect CNS^15^.

The immunopathological changes observed in murine leprosy at the late stages of the disease are driven by Th2 anti-inflammatory cytokines that facilitate progression of the disease in a manner similar to what happens in lepromatous leprosy^7^. Like in multibacillary leprosy patients^31^, we found coexistence in serum of IL-12 and IL-4 on day 80 PI, and also the presence of these cytokines in the liver and spleen of *MLM*-infected animals. The increase in the levels of IL-4 has been related to CMI anergy that develops as the infection progresses^32,33^, this observation coinciding with our results of high bacillary load and positive immunostaining for IL-4 in liver and spleen. The T-maze test was used to evaluate alterations in working memory along the infection^34^. Our results showed a significant increment in the latency-time that correlated with longer times of infection, reduction in the number of successful attempts, and increment in the time required to fulfill a successful event^35^. These results indicated a significant loss in the capacity to accomplish an activity associated to working memory and are in agreement with previous results that demonstrate that administration of LPS or bacteria deteriorate this brain activity^36–40^. The mechanism responsible for this brain deterioration has yet to be elucidated, but the neuroinflammatory effect of persisting high levels of cytokines is a likely cause of it^4,5,41,42^. These alterations have also been documented in models of non-infectious chronic stress^43–45^. Using the spontaneous locomotor activity test we excluded the possible existence of lesions in other regions of the brain as a result of the infection^26,27^. Contrastingly, the only alterations found were located in the hippocampus (Figs 4 and 5). Because our findings are based on a limited number of evaluated regions, the results should be interpreted with caution. Systemic chronic infections have repercussion on the CNS activity^3–5^ and the persistent peripheral stimuli might lead to production of neurotoxic molecules in the brain, causing neuroinflammation^3^. Therefore, we searched for parenchymal alterations in the dorsal region of the hippocampus considering that it is a target region for infectious and non-infectious chronic stress^46–48^. We found pyknotic/acidophil neurons in the CA1, CA2, CA3, and dentate gyrus (DG) regions along with the progress of the infection. This is an important feature considering that the dorsal region of the hippocampus plays a key role in learning and memory processes^49–51^. Additionally, CA1, CA3, and DG constitute the classic tripartite-synapse pathway, an important route for memory processes^52^. While inflammatory stimuli, such as systemic LPS administration, impaired working memory affecting CA1, CA3, and DG regions due to ROS-mediated neuroinflammation^46^, CA3 deterioration altered the synaptic transmission to CA1 and the completion of memory processes^47,53^. On the other hand, the neuronal loss in DG is relevant because this region is involved in the early development of spatial memory and the production of new neurons along life^54,55^. Finally, the CA2 region participates in social memory and its deterioration affects recognition, spatial, and contextual memory^56,57^.

Morphological alterations were concomitant to changes in neurotransmitter levels, which are crucial in the optimal function of brain processes including learning and memory^58–61^. During infection, 5-HT and DA levels in the hippocampus of infected mice were lower than in the hippocampus of uninfected animals. The decrease of these neurotransmitters in the hippocampus has been shown to be associated to memory deficit, neuronal atrophy, and neuronal loss in a model of hepatic encephalopathy^59^. Also, memory and learning impairment has also been reported after the injection of 6-hydroxy-dopamine (6-OHDA) which selectively induces dopaminergic and noradrenergic neuron neurotoxicity^60^.

## Conclusion

This work refers to a murine leprosy model in which the disease does not directly affects the nervous system but is, however, the cause of alterations in the working memory, the morphology of the dorsal region of the hippocampus, and the levels of neurotransmitters. We regard that murine leprosy as an infection model suitable for further exploring the effects of chronic infections on the structure and function of the central nervous system.

## Materials and Methods

### Mice

Forty male BALB/c mice, 12–14 weeks old, purchased from Harlan, Mexico, were properly housed in polypropylene cages and fed Harlan chops and purified water *ad libitum*. They were kept at a constant temperature (23–24 °C), under 12 × 12 h light-darkness cycles. Handling of the animals was performed under the standards approved by the Research Ethics Committee of the National School of Biological Sciences, under the code CEI-ENCB016/2014.

### Infection with *MLM*

*Mycobacterium lepraemurium* (*MLM*) is a hardly cultivable mycobacterium, resembling *Mycobacterium leprae* in this aspect. Therefore, *MLM* (Hawaii strain) was isolated from liver and spleen of a mouse bearing a 4-month infection, as described elsewhere^62^. Viability of bacteria was assessed using the fluorescein diacetate-ethidium bromide method by Jarnagin *et al*.^63^, as previously described^62^.

A group of 30 mice was intravenously inoculated with 20 × 10^06^ *MLM* in 50 µL saline solution. Control mice only received saline solution. Mice were sacrificed at the same time of the day to avoid circadian variations.

### Histologic and morphometric analysis

Mice of the control group and those infected for 40, 80, and 120 days were euthanized by CO_2_ inhalation and immediately heart-perfused with 10% neutral formalin. After 10-min perfusion, the brain, spleen, and liver were collected and preserved in the same fixer solution for 5 days. Then, the organs were trimmed and prepared for paraffin sectioning according to standard procedures. Three-micron thick sections were obtained using a Leica microtome, mounted on poly-L-lysine-coated slides, and stained with hematoxylin-eosin (H&E) for conventional histology, and Ziehl-Neelsen (ZN) stain for acid-fast bacilli. Images were taken in a Nikon Eclipse E8000 microscope (Tokyo, Japan). Bacillary density in the liver and spleen was quantified using Image J software and the results were given as red pixels^12^.

### Measurement of cytokine levels in serum

Sera were collected by cardiac puncture, using Microtainer tubes with separation gel. Serum cytokines were measured using a multiplex kit (Bead Mouse Th1/Th2 6-Plex Panel, Invitrogen, Multi-Cytokine Detection System) for IL-2, IL-4, IL-5, IL-10, IL-12, and IFN-γ in a Luminex® LABScan 100 apparatus, according to the manufacturer’s protocol.

### Determination cytokines by immunohistochemistry

For immunohistochemistry, 4-µm thick sections of liver and spleen were prepared and collected on poly-L-lysine-coated slides. After deparaffination and quenching of endogenous peroxidase with 3% hydrogen peroxide solution and methanol by 30 min, sections were incubated with rabbit anti-IL-4 and IL-12 antibodies (Santa Cruz Biotechnology diluted 1/200 in PBS by 3 hrs at room temperature and subsequently incubated with goat anti-rabbit IgG-peroxidase diluted 1/500 for 30 min at room temperature. After three washings with PBS-Tween, sections were stained with haematoxylin, mounted with synthetic resin, and examined under the microscope. Images were taken in a Nikon Eclipse E8000 microscope (Tokyo, Japan).

### Working memory

We decided to use the T-maze to reduce the stress generated by the averse stimuli employed in other mazes^64,65^. Working memory was evaluated by a standard protocol using a stainless-steel T-maze (OMNIALVA, Instruments, Mexico) consisting of three sections: a left and a right arm (75 × 20 × 40 cm each) and a starting box (30 × 25 × 30 cm). A small circular pot (6-cm diameter and 3-cm depth) was placed at the end of each arm. A food reward (0.5-g, Test-Diet pellet, chocolate flavor) was placed in the pot and could not be seen or smelled by the mouse from the start box. The working memory test was used to evaluate hippocampus-dependent memory^32^. For the habituation process, each mouse was placed in the T-maze after a 24 h fasting period. Mice were laid in the maze for 30 min and allowed to move freely to the aisle of their choice; they eventually found the sweet (award). Two days later, each animal was subjected to 12 trials of rewarded alternation in the T-maze. Mice were allowed a maximum of 120 sec to complete a trial. The T-maze allowed us to evaluate the latency time, percentage of success, and time for a success.

### Spontaneous locomotor activity

To estimate the spontaneous locomotor activity, each mouse was placed in a Plexiglas cage (50 × 50 × 30 cm) surrounded by a set of 16 light-beam photocell pairs appropriately positioned to register the number of light interruptions resulting from the animals’ movement^66,67^. The light registered by the photocells was transformed into digital pulses, which were then sent to a desktop computer. Data were processed and analyzed by means of OABiomed software. Animals were adapted to the activity chamber for three sessions of 30 min each, and then they were individually placed into the chamber and monitored for 30 min.

For the behavioral tests, each group was randomly divided into two subgroups, each with five mice. The first group was used for the T-maze test and the second for the spontaneous locomotor activity test. In these experiments, working with five mice allowed us to avoid the stress induced by the fasting period and exposure to an open field in the spontaneous locomotor activity test.

### Histologic and injury assessment in the hippocampus

Mice were euthanized by CO_2_ inhalation and immediately hart-perfused with isotonic saline solution for 5 min and then with 10% neutral formalin. After a 10-min perfusion, brains were removed and included in brain matrix to a 1.70 mm bregma depth. Then, the brains were trimmed and prepared for paraffin sectioning according to standard procedures. For the morphometric analysis, 3-micron thick sections were collected on poly-L-lysine-coated slides and stained with the H&E and ZN stains. To investigate the presence of bacilli (*MLM*) in the CNS, brain sections were stained with the ZN stain. Five pictures from each section corresponding to the hippocampus dorsal region (bregma coordinates −1.82 and −2.46) were taken (Nikon Eclipse E8000 microscope,Tokyo, Japan). Five microscope fields of regions CA1, CA2, CA3, and DG were analyzed to calculate the number of cells showing morphological alterations.

### Neurotransmitter quantification by HPLC

To quantify serotonin (5-HT), dopamine (DA), norepinephrine (NE), and epinephrine (EP), the hippocampus was homogenized using 400 µl of a solution containing 5% ascorbic acid, 200 mM sodium phosphate, 2.5 mM L-cysteine, and 2.5 mM EDTA. Then, the protein was precipitated by the addition of 100 µl of 0.4 M perchloric acid, followed by incubation at 20 °C for 20 min. Supernatants containing NE, EP, DA, and 5-HT were collected after centrifugation at 12,000 rpm for 10 min (4 °C). NE, EP, DA, and 5-HT concentrations were determined by reversed-phase HPLC (RP-HPLC) in a system integrated by two 515 pumps (Waters™), a degasser AF (Waters™), a 717 autosampler (Waters™), and an X-LC™3120FP fluorescence detector (Jasco, Inc). Instruments were controlled by Millenium 32 software (Waters™). Chromatographic runs were performed using a Jupiter C18 column (300 Å, 5 μ, 4.6 × 250 mm, Phenomenex®) at 30 °C. Column was equilibrated with the mobile phase A (MPA) containing 0.1% trifluoroacetic acid. Mobile phase B (MPB) containing 0.1% trifluoroacetic acid in acetonitrile was used to perform a linear gradient until reaching 20% MPB, from min 5 to min 15. Then, 20% MPB was maintained until min 20; the flow rate was 0.8 ml/min. The fluorescence detector was set at gain 100, attenuation 32, response 20 s, and 280 nm and 315 nm for excitation and emission, respectively. The sample injection volume was 50 μl.

### Statistical analysis

Results are presented as the average mean value ± standard error of mean (SEM). Data for bacillary load, cytokine and neurotransmitter levels, hippocampus cell injury, and memory loss were analyzed by one-way ANOVA and Tukey’s test for multiple comparison. Graphs were produced by using GraphPad Prism 4 software. A difference was considered significant when p < 0.05.
